# Trends in Coffee and Tea Consumption during the COVID-19 Pandemic

**DOI:** 10.3390/foods10102458

**Published:** 2021-10-15

**Authors:** Fabio Castellana, Sara De Nucci, Giovanni De Pergola, Martina Di Chito, Giuseppe Lisco, Vincenzo Triggiani, Rodolfo Sardone, Roberta Zupo

**Affiliations:** 1 Unit of Data Sciences and Technology Innovation for Population Health, National Institute of Gastroenterology “Saverio de Bellis”, Research Hospital, Castellana Grotte, 70124 Bari, Italy; fabio.castellana@irccsdebellis.it (F.C.); sara.denucci@irccsdebellis.it (S.D.N.); zuporoberta@gmail.com (R.Z.); 2 Unit of Geriatrics and Internal Medicine, National Institute of Gastroenterology “Saverio de Bellis”, Research Hospital, Castellana Grotte, 70124 Bari, Italy; gdepergola@libero.it (G.D.P.); martinadichito1999@gmail.com (M.D.C.); 3 Section of Internal Medicine, Geriatrics, Endocrinology, and Rare Disease, Interdisciplinary Department of Medicine, School of Medicine, University of Bari, 70124 Bari, Italy; g.lisco84@gmail.com (G.L.); vincenzo.triggiani@uniba.it (V.T.)

**Keywords:** dietary behavior, COVID-19, beverage consumption, coffee, tea

## Abstract

Over the last two years, many countries have enforced confinement to limit both the spread of COVID-19 and the demand for medical care. Confinement has resulted in a disruption of work routines, boredom, depression, and changes in eating habits, among them consumption of coffee and tea. Following six databases, we examined articles tracking consumption of these beverages. Out of 472 articles, including 23 beverage entries, 13 matched our criteria. While no clear trend in coffee consumption during the coronavirus pandemic emerged (7 of 13 studies indicated an increase, accounting for 53.8%), tea consumption clearly increased (70% versus 30%). Considering the global health emergency continuum, more research is needed to better understand the paths underlying food choices and the ways those changes may influence health outcomes, including those related to COVID-19 disease.

## 1. Introduction

The coronavirus pandemic still poses a worldwide public health challenge, with 216,229,741 cases confirmed since the World Health Organization (WHO) declared a state of global emergency in March 2020 [1]. At present, Europe is experiencing increased infections due to social mixing, summer travel, family reunions, and looser social restrictions. Set against this backdrop, a recent report by the WHO director for Europe warns of three conditions that could contribute to a new wave of hospitalizations and excess deaths before the fall: new variants, incomplete vaccine adherence (63% of people are still reluctant to undergo vaccination), and increased social mixing. By way of prevention, most governments have imposed varying degrees of self-isolation and nationwide lockdowns to curb spread of the virus. Staying-at-home has meant digital education, smart working, social isolation, job insecurity, and limited outdoor and gym activity-in short, a dramatic change in lifestyles [2]. Moreover, quarantine and distancing from families have led to a cluster of negative psychological implications, including confusion, anger, and depression due to frustration, boredom, inadequate information, and financial loss [3,4]. This burden of unpleasant feelings, combined with limitations at multiple levels, has prompted substantial changes in lifestyle, triggering a shift in eating habits in terms of a reduced control over food intake and quality. These changes include escalations in carbohydrate intake, the frequency of snacking, and home cooking during confinement [5]. Often home cooking entails a higher consumption of homemade cakes, bread, and pizza, all sharing a critical glycemic load, which could affect weight. In most cases, people ate “comfort” foods to reduce accumulated stress, relying on the biological effect of serotonin on mood.

Beverage choices also contribute to daily calorie intake and hydration, particularly nervine beverages such as coffee and tea, in view of their known potential to promote psychological well-being beyond the mere nutritional aspect [6]. Tea affects psychopathological condition (e.g., reduced anxiety), cognition (e.g., benefits in memory and attention), and brain function (e.g., activation of working memory). Yet such benefits are not attributable to a single constituent, and better gains yielded by the synergy of caffeine and L-theanine are reported as compared to their separate administration [7]. As for coffee, much scientific attention has been paid to its association with mood and emotion. One cup of coffee every four hours improves mood. Low to moderate doses of caffeine (two to five cups of coffee per day) have been shown to improve hedonic tone (the degree of pleasantness or unpleasantness associated with a given state) and reduce anxiety [8,9]. 

Against this background, it seemed useful to further probe the influence of COVID-19 confinement on consumption of these beverages in order to consider possible health implications, related not only to mental well-being but to overall health. 

## 2. Methods

The present work is a narrative review article. We searched the US National Library of Medicine (PubMed), Medical Literature Analysis and Retrieval System Online (MEDLINE), EMBASE, Scopus, Ovid, and Google Scholar to find original articles covering dietary variables during the COVID-19 pandemic, selecting studies examining changes in coffee and tea consumption (see Table 1). Given the novelty of the topic and the short timeframe surrounding COVID-19 pandemic research, no skimming was applied to the study population, design, or setting. For the same reason, no age range was applied to the study population. Two investigators (RZ, SD), independently and in duplicate, searched for papers, screened titles and abstracts of the retrieved articles, reviewed entire texts, and identified articles for inclusion in this study. Of particular interest were original articles investigating dietary habits, particularly coffee and tea consumption, during COVID-19 via online or telephone questionnaires and those utilizing accounts of weekly or monthly food intake. Of note, reports offering a snapshot of coffee and tea consumption during the period of COVID-19 confinement were excluded in favor of studies comparing the consumption of the two beverages before and after the COVID-19 pandemic. The investigators tracked (1) such general information as study design, setting, sample size and demographics (age and gender), country, method of dietary assessment, and dietary exposure; and (2) principal results of nutritional surveys (changes in trends and frequency of coffee and tea consumption). Data were reported separately for coffee and tea consumption with respect to increase or decrease. Data were cross-checked by a third researcher (FC) to remove discrepancies and resolve disagreements. 

## 3. Results

**Figure 1:**
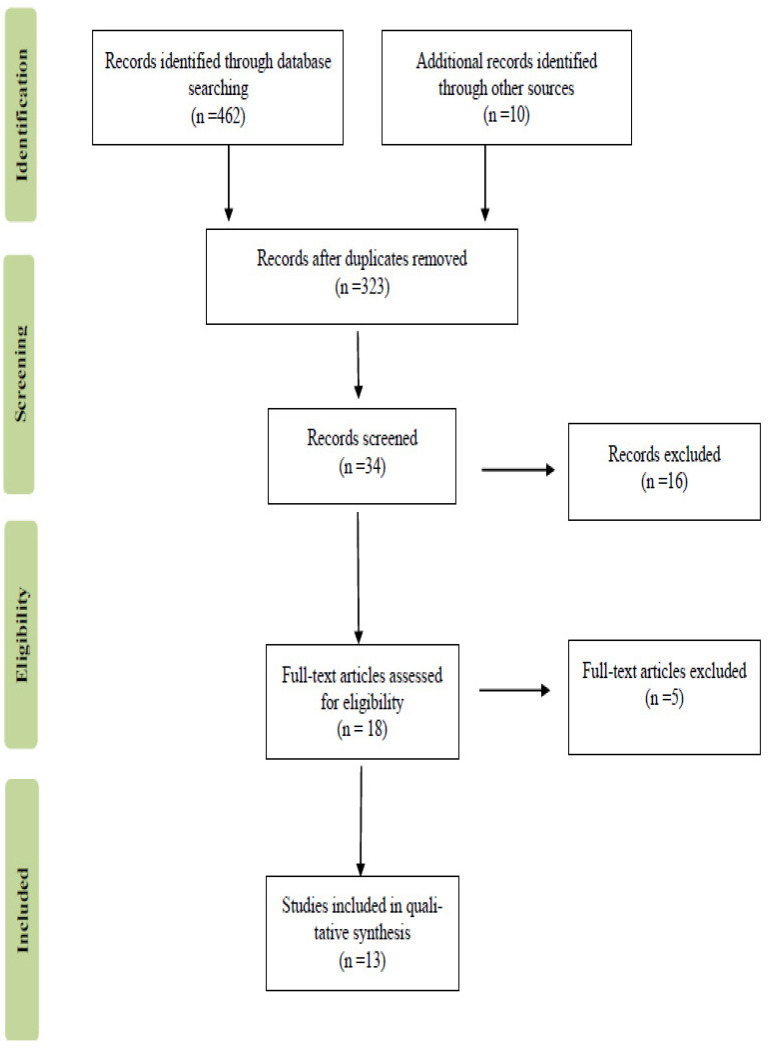
Flow diagram of literature screening process.

The search strategy was updated to 31 August 2021, and yielded 472 results. Of these, 13 were found pertinent to our objective and selected for analysis [10,11,12,13,14,15,16,17,18,19,20,21,22]. Figure 1 shows a flowchart of the literature screening process. Each of these 13 reports utilized online or telephone questionnaires about dietary habits covering a cross-section of the population. All studies were based on community sampling, and respondents were older than eighteen years. Overall, these studies included 435,616 subjects, more prevalently female (70.2%, *n* = 305,802 versus 29.8%, *n* = 129,816). The majority of the studies were European (9/13, 70%), with a minority of American and Asian (2/13, 15% each). Only one report had a multicenter setting [18], reporting data from Poland, Austria, and the UK. Table 2 covers details of the design (cohort or cross-sectional), sample size (*n*) and gender ratio (%), minimum age and mean (SD) or age range, setting (community or hospital), and country.

Though only 10 of the 13 selected studies investigated both coffee and tea as beverages, we analyzed a total of 23 beverage entries. It should be noted that 7 of these 10 studies considered the combined consumption of both tea and coffee, rather than treating each individually. Of the 23 entries, 13 considered coffee consumption (7/13, *n* = 54%) and the remaining 9, tea (9/13, *n* = 46%). As regards coffee, 54% of studies indicated an increase, while the remaining 46% reported a decrease in consumption. By contrast, in 70% of studies (7 or 10) tea consumption was reported as having increased while in 30% of studies (3 of 10) is was reported as having decressed. Only one report analyzed coffee consumption by variety, i.e., whether American or Arabic [11].

## 4. Discussion

We reviewed existing literature on changes in coffee and tea consumption driven by confinement during the COVID-19 pandemic with respect to coffee and tea consumption, in view of their known impact on psychological well-being. We found no clear trend in coffee consumption, while there was a clear increase in tea consumption. However, looking at coffee consumption, it should be considered that our methodological setting only included original reports comparing the period of confinement to the previous time. However, based on the literature search, we found a survey from Poland reporting the highest frequency of coffee consumption (88.9%) among adults aged 45+ but referring only to COVID-19 confinement, with no comparison to previous habits [23]. The high frequency of coffee consumption recorded in this study suggests an increasing consumption of this beverage during the pandemic.

The unclear findings on increased coffee consumption may be understood from both social and psychological perspectives. On the one hand, people who are used to drinking coffee in family contexts on a daily basisalso enjoy coffee in social situations. Especially among adolescents, drinking coffee is a way to spend time with friends and improve one’s mood [24,25]. Moreover, compared to adults, adolescents are particularly oriented toward the upgraded social image they can project by consuming caffeinated beverages. Such an aspect, if read in a pandemic key, would imply a drop in consumption, as social and community events were curtailed during COVID-19 confinement. On the other hand, from a purely psychological and emotional point of view, coffee is a good source of energy and may be used to improve mood, fight drowsiness, and enhance cognitive function [26,27,28]. This second point, read in a pandemic key, could explain increased coffee consumption, in light of the widespread smart working scenario and the distressed mood caused by the pandemic itself.

As for consumption of tea, findings suggest a clear increase in consumption compared to tea drinking before COVID-19.Tea is usually linked to routine and ritualized household consumption. Tea is historically instrumental in bringing the family closer together and provides a platform for sharing. In contrast, coffee consumption needed to be considered in a social, aesthetic and emotional context. Setting aside the social context, therefore, the increased consumption of tea should be understood in emotional and family-related settings. From this perspective, this beverage It has long been associated with mood and performance enhancements, such as a greater relaxation and concentration. Though tea contains many bioactive compounds, but its benefits with respect to attention, mood, and the cognitive sphere have generally been attributed to two of its components, namely, caffeine and theanine [29]. A cup of tea provides 35–61 mg of caffeine and 4.5–22.5 mg of theanine. A substantial body of research suggests that L-theanine exerts anti-stress effects in response to acute stress challenges via the inhibition of cortical neuron excitation. On the other hand, caffeine found in coffee has been reported to improve performance and memory, reduce errors in performing tasks, accelerate cognitive processing, and improve mood [30,31]. Moreover, caffeine improves concentration and attention by eliminating distractors and improving focus, which is the reason why it has the potential to improve vigilance and reaction time [8]. Yet, in pandemic settings, this feature may be read as a driver of increased tea consumption, driven by higher levels of stress and confusion induced by the epidemic situation and the dissemination of home-based smart working [32].

## 5. Conclusions

The lack of a clear trend in coffee consumption as the result of the COVID-19 pandemic calls for further investigation. Moreover, potential health implications should not be overlooked, especially since caffeine consumption may directly or indirectly promote bronchodilation, interfere in the process of immunomodulation, and hinder viral intracellular transcription while undergoing COVID-19 infection [33]. Furthermore, reflecting a discomfited mood and socially confining setting, we found a marked increase in tea consumption.

## 6. Limitations

More studies are needed to expand on these findings and examine coffee and tea consumption separately. The limited number of studies included is a major limitation of the present review, weakening the completeness and generality of findings, despite their being quite representative of the European population. Secondly, some of the selected studies examined the combined consumption of tea and coffee, rather than taking each individually. Nevertheless, this preliminary research provides food for thought. Lastly, though coffee and tea stand out as the most popular beverages worldwide, we know the cluster of nervine beverages is much wider, including cocoa, cola drinks, guarana, and maté, all of which have a tonic and stimulating effect on the central nervous system, due to the presence of natural alkaloids (for example caffeine, theophylline, theobromine, etc.) 

## Figures and Tables

**Table 1:** Search strategy to be used in the US National Library of Medicine (PubMed) and Medical Literature Analysis and Retrieval System Online (MEDLINE) and adapted to the other sources, according to selected descriptors.

Strategy	Descriptors Used
# 1	(diet*[tiab]) OR (feeding*[tiab])) OR (habit*[tiab]) OR (dietary lifestyle*[tiab]) OR (drinking habit*[tiab]) OR (beverage*[tiab]) OR (dietary habit*[tiab]) OR (dietary [tiab]) OR (dietary pattern*[tiab]) OR (dietary behavior*[tiab]) OR (food*[tiab]) OR (food habit*[tiab]) OR (eating habit*[tiab]) OR (coffee[tiab]) OR (tea[tiab])
# 2	(change*[tiab]) OR (modification*[tiab]) OR (alteration*[tiab]) (different*[tiab]) OR (difference*[tiab])
# 3	(SARS-CoV 2[tiab]) OR (COVID 19[tiab]) OR (severe acute respiratory syndrome coronavirus 2[tiab])
# 4	(Review) or (systematic review) or (narrative review) or (meta-analysis)
# 5	#1 AND #2 AND #3 NOT #4

**Table 2:** Descriptive characteristics of included studies.

Authors, Year [Ref.]	Diet Exposure	Diet Assessment Tool	Design	*n*	Sex	Age	Setting	Country	Results	Summary of Findings
Bin Zarah A et al., 2020 [10]	Coffee, Tea	Questionnaire	Cross-sectional	3133	19.8%M 79.4% F	18+ years	Community	America (USA)	About 10% declared a reduction in frequency of consumption, whereas 31.1% an increase.	Participants reported a higher consumption of coffee and tea drinks.
Husain W et al., 2020 [11]	Coffee, Tea			415	31.3%M 68.7%F	18+ years		Asia (Kuwait)	1–2 cups/day of American coffee: 41.4% (before) vs. 33% (during) 3–4 cups/day of American coffee: 8.7% (before) vs. 5.5% (during) 1–2 cups/day of arabic coffee: 15.4% (before) vs. 15.5% (during) 5–6 cups/day of arabic coffee: 10.6% (before) vs. 7.7% (during) 1–2 cups/day of tea: 31.6% (before) vs. 33% (during) 5–6 cups/day of tea: 1.7% (before) vs. 2.9% (during)	Decreased consumption of American and Arabic coffee during the confinement, versus increase in tea consumption.
Błaszczyk-Bębenek E et al., 2020 [12]	Coffee, Tea			312	35.9%M 64.1%F	18+ years		Europe (Poland)	Hot beverage consumption frequencies: 1–3 times/month: 3.2% (before) vs. 3.5% (during) Once a week: 1.3% (before) vs. 1.6% (during) A few times a week: 5.1% (before) vs. 7.1% (during) Once a day: 16.0% (before) vs. 16.3 (during)	Hot beverages such as black coffee, herbal, or fruit tea were chosen most frequently by respondents during the confinement.
Grabia M et al., 2020 [13]	Coffee			124	17%M 83%F	17–45 years		Europe (Poland)	30% reported an increase in frequency of coffee consumption while 13% reported a reduction.	Increased coffee consumption during the confinement.
Sánchez-Sánchez E et al., 2020 [14]	Coffee, Tea			1073	27.2%M 72.8%F	16+ years		Europe (Spain)	Frequency of coffee or tea consumption: 7.89% (before) vs. 6.48% (during)	Decreased coffee or tea consumption during the confinement.
Đogaš Z et al., 2020 [15]	Coffee			3027	20.3%M 79.7%F	18+ years		Europe (Croatia)	Frequency of coffee consumption (cups/day): All participants: 2.1 ± 1.0 (before) vs. 2.1 ± 1.1 (during) Males: 2.4 ± 1.2 (before) vs. 2.0 ± 1.2 (during) Females: 2.1 ± 1.0 (before) vs. 2.1 ± 1.1 (during)	Croatian males drank fewer cups of coffee during confinement.
Di Renzo L et al., 2020 [16]	Coffee, Tea			3533	23.9%M 76.1%F	12–86 years		Europe (Italy)	Hot beverage consumption frequency increased by more than 20% during confinement.	Greater than 20% increase in the consumption of hot drinks
Luo Y et al., 2021 [17]	Coffee, Tea			2272	18.3%M 83.4%F	18+ years		Asia (China)	Coffee, tea, and water consumption increased by 29.3%.	Hot beverage consumption increased during confinement.
Skotnicka M et al., 2021 [18]	Coffee, Tea			1071	43.6%M 56.4%F	18+ years		Europe (Poland, Austria, UK)	Frequency of coffee consumption (cups/day): Poland: 76.90% (before) vs. 76.17% (during) Austria: 62.61% (before) vs. 62.32% (during) UK: 54.34% (before) vs. 49.84% (during) Frequency of tea consumption (cups/day): Poland: 57.74% (before) vs. 60.93% (during) Austria: 54.11% (before) vs. 58.36% (during) UK: 64.31% (before) vs. 74.59% (during)	More frequent consumption of tea and less frequent consumption of coffee during confinement.
Celorio-Sardà R et al., 2021 [19]	Coffee, Tea			321	20.2%M 79.8%F	18+ years		Europe (Spain)	There was a decrease in coffee and tea consumption during confinement reported by 56.7% of the study sample.	Decreased coffee and tea consumption during confinement.
Izzo L et al., 2021 [20]	Coffee			1519	28.4%M 71.6%F	0+ years		Europe (Italy)	Coffee consumption increased for 64.8% of participants.	Increased coffee consumption during confinement.
Mitchell E.S. et al., 2021 [21]	Coffee			381,564	16.6%M 83.4%F	18+		America (USA)	Caffeinated beverages such as tea and coffee decreased in the proportion of users aged 18–35 years (−2.3%), but only marginally decreased in users aged 35 years and older.	Decreased tea and coffee consumption during confinement.
Deschasaux-Tanguy M et al., 2021 [22]	Coffee, Tea			37,252	47.7%M 52.3%F	18+ years		Europe (France)	Frequency of tea consumption during confinement: Increased for 19.5%, for decreased 4.3%. Frequency of coffee consumption during confinement: Increased for 13.5%, decreased for 8.4%	Increased coffee and tea consumption during confinement.
